# Atlanta Academy of Medicine

**Published:** 1875-11

**Authors:** 


### Atlanta Academy of Medicine.

JAMES B. BAIRD, M.D., Reporter.

Atlanta, September 21, 1875.

The President, Dr. J. M. Johnson, presiding.

Dr. H. F. Scott read the following essay on dysentery, as observed during May and June of the present year, with the mode of treatment:

"During the above months it was my lot, as City Physician, to encounter a great many cases of dysentery. This was, doubtless, the experience of most members of the academy. Indeed, so prevalent did it become that I was told by some of the oldest and most extensive practitioners of the city that they had not met with so many cases since the epidemic of 1871. In fact, so common was it in my practice during these months to treat dysentery that I was seldom called to treat any other disease, and for two successive weeks of the time, although I made several calls during each day, yet I saw nothing but it. Nor was it confined to any age, but all alike had it, from the youngest infant at its mother's breast to the gray haired old man.

"But whilst it was extensive, yet at the same time it was mild, and yielded kindly to treatment. Indeed, several cases under my supervision got well without any treatment, other than dieting, in the course of a week; whilst the remainder, of somewhat more serious a type, under a course of treatment which I shall hereafter describe, almost invariably recovered within from two to four days, and in several instances after taking only one dose of medicine—in this respect differing from the aforesaid epidemic, which I am informed was unusually difficult to manage.

"In treating these cases I used, in some fifteen of them, ipecac according to Dr. Woodhull's manner of treating them, and with pretty fair success; upon others I used various plans of treatment, with varying success; but towards the last I relied altogether upon Epsom salts and laudanum, in some cases giving a full dose of salts with ten drops of laudanum, to be followed by several doses of laudanum alone, which answered well in some cases; but in the majority of cases I would put, say a tablespoonful of salts and a teaspoonful of laudanum in a wineglass of water, and direct a teaspoonful to be given every half hour. In this manner getting the depleting and cooling effect of the salts, together with the soothing action of the laudanum, and at the same time a cleaning of the bowels of any irritating substances that may be present.

"I am aware that this is an old treatment, but at the same time it is my opinion, based on the above experience, together with my former and subsequent experience in connection with this disease, that the above treament, with the application in some cases of warm poultices to the abdomen and appropriate dieting, is the most rational of any, and at the same time the most effective treatment for this disease."

Dr. W. S. Armstrong reported seven cases of scarlatina, all having occurred in the last few weeks.

Dr. Woodhull inquired if it was a fact that scarlatina was invariably of a mild type in this latitude?

Dr. J. G. Westmoreland said he had not seen a case of scarlet fever for eighteen years. Eighteen years ago had three cases, widely separated, in this city—one died. Thinks the skin is always smooth. The eruption is not papular. The epidemic of 1843 was very fatal.

Dr. Logan remarked that the eruption of scarlatina was not simply an efflorescence but slightly elevated, and the elevation being uniform, is not very distinct. The history of this affection teaches that it is contagious, but it is an uncertain contagion. His observation confirming this position.

Dr. J. G. Westmoreland observed that his reading teaches him scarlatina is contagious, but his experience is not in accord with the books. Saw no evidence of contagion in the epidemic of 1843.

Dr. Logan added that Louis Thomas, (Ziemssen's Encyclopedia, vol. ii., p. 175,) explains the apparent uncertainty of the contagion, on the ground that a considerable number of persons are not susceptible to its influence.

Dr. J. M. Johnson said in the eruption of scarlatina the skin is smooth, except the thick skin. In certain situations it may be papular.

Dr. Armstrong closed the discussion on this subject, saying:

"I was induced to write a report of the above cases for the reason that there seems to exist a doubt in the minds of many here that scarlatina is prevailing in this place. Though the number of cases seen by me has not been great, (probably not more than twenty,) I have been satisfied of its existence here since last winter.

"Since I have been in the practice, there has been no epidemic of this disease in my region of country, and my knowledge of it, clinically, is confined to seeing it treated in London several years ago. There I was told it did not then prevail as an epidemic, but that the city was never free from it. The cases witnessed at that time were sufficiently abundant for me to become thoroughly familiar with it in its various phases.

"In 1872 I treated a case that occurred near this city, which was also seen by Drs. Bozeman and Logan. This is the only case I remember to have seen here before the present occurrence of the disease.

"Because it is not as contagious as measles and small-pox, and does not sweep through the community and destroy its victims by the hundred, as epidemics of other diseases have done, is certainly no argument against its prevalence. Besides, we read of epidemics so mild that most of the cases are not confined to bed.

"While it is the generally received opinion that it is not so contagious as measles,—and my experience coincides with this view,—I have seen it carried from one house to another by persons visiting. Another fact is also made very clear by the cases I have seen, viz: that children under ten years of age are much more liable than those older to contract scarlatina. From the cases reported, it will also be apparent that, in the same families, the severity of the affection is mainly confined to children; and this has been my observation among my other cases.

"I have only seen one adult who had the eruption even in a light form. Yet all the other symptoms were present.

"Of the seven cases reported, all four of the children had the eruption, inflammation of the tonsils, redness of soft palate, anterior palatine arches, and lymphadinitis, together with cellulitis. On the other hand, the three adults had all of these symptoms, except the eruption. Yet it will hardly be doubted, because the eruption was absent, that the disease was not the same as the children were suffering from. It would be equally unfair to say that it was not scarlatina because they were not very ill.

"It has been said that some symptoms of scarlatina were absent in my cases. I would be glad to know what they are. The well marked cases have all had the characteristic punctiform eruption going through its various stages over the body, the angina, the characteristic appearance of the tongue, inflammation of the cervical lymphatics as well as of the connective tissue of the neck.

"There seems to be no symptom wanting to constitute this disease, scarlatina. But should there still be a doubt, after this array of symptoms, it seems as if it should have been dispelled later when the desquamation came on, and when acute desquamative nephritis, followed by general and local dropsy, had also made its appearance. The desquamation has been witnessed in all the cases, and nephritis followed by dropsy in three children and one adult within the last two months.

"In reply to the President, Dr. Johnson, who states that my description of the punctate elevated points differ from the smooth rash he met with many years ago, I reply, in the language of Thomas, of Leipzig, that the exanthem of scarlatina "has been variously described by different authors, undoubtedly because its character varied at different times and in different places, a manner corresponding to the variation of the entire disease." And further on, he says: "They" (the punctate eruption) "are either entirely flat, or, as is more often the case, very slightly elevated, but not near as much so as in the roseola of measles. * * In order to cause a general confluence of the eruption, but little additional growth of the single points is necessary; in severe cases, in which the exanthem is of longer duration, confluence very often occurs, which accounts for the frequent description of scarlatina as a hyperæmia that is uniformly distributed over the whole body." Hence, I consider the objections of the President to the difference in appearance of the eruption, as seen by me here, from that witnessed by him many years ago, fully answered by the description of the variations in the eruption given by Thomas, as seen at different times and in different places in various epidemics."

Dr. Taliaferro desired to call the attention of the Academy to a communication read before the Middle Georgia Medical Society, and published in the October number of the ATLANTA MEDICAL AND SURGICAL JOURNAL, upon "Anæsthesia in Labor," by Dr. K. P. Moore, in which the following language occurs: "* * * It may seem presumptuous in one of my age to attempt an elucidation of a subject which has been handled by men of long experience, and extended observation and research; but I do not wish to be understood as assuming to myself any superior knowledge upon this subject, nor of presenting any thing new to the gentlemen of the Academy, or Dr. Washington, but of calling attention to what they seem to have overlooked, and which is necessary to strengthen Dr. Washington's paper, as also Drs. J. M. Johnson, Taliaferro, and others, who have *timely confessed* (italics ours) that they could be persuaded under very trying circumstances to use chloroform in labor, (the verdict of a Boston jury to the contrary notwithstanding.)"

A little further on Dr. Moore says: "Of course every condition, which would contra-indicate its use, should be looked well into, and *exceeding caution observed. I always watch its effect upon the circulation and respiration especially.*"

In the discussion referred to, Dr. J. M. Johnson used the following language: "Has heard a great deal said about the dangers of chloroform, but I think with Sir James Simpson, that a man who will not use it in obstetrical cases, is almost a heathen. It may be given in a manner free from danger, and productive of great good," etc.

Dr. Taliaferro said he "is impressed with the danger of going to an extreme in abandoning the use of chloroform through fear of its effects; uses it in labor when he deems it advisable. The intensity of the pain is moderated. Never knew of a death from chloroform in obstetrical practice, and, so far as his knowledge extends, none had been reported. In surgery prefers ether; considers it safer. Chloroform is preferable in obstetrics, as it acts quicker, and to secure its effects it is not necessary to saturate the patient with it."

Dr. J. M. Johnson further remarked that "he invariably gives chloroform in labor, always in small quantity, and always with the same result."

Dr. Taliaferro said in reply to the President, he would state that until the last few years he was in the habit of using it freely to complete anæsthesia in almost every case. Never saw a case in which the uterine contractions were retarded, or the action of the abdominal muscles suspended. Does not give it now to the same extent he formerly did; not because he is not an advocate of it, he is, but on account of the big scare the profession are in about chloroform, fearing some accident may occur. If his patient requests chloroform she gets it. Has never seen the least unpleasant effect from it, either to the mother or child. Should now use it to anæsthesia if necessary."

Thus it appears that Drs. Johnson and Taliaferro administer chloroform boldly, but cautiously, while our friend Dr. Moore is evidently himself *timid* about the "circulation and respiration especially."

R. W. WESTMORELAND, M.D., Reporter.

Atlanta, September 28, 1875.

Academy met, Dr. John M. Johnson, President, in the chair. Report of cases being in order, Dr. W. F. Westmoreland called attention to the case of a two year old colored female child presented at his office last Saturday. On investigation, it was found that there had been retention of urine for twenty-four hours, and that the labia minora were adherent to a point about the orifice of the urethra, leaving, however, an aperture at the upper portion, through which a probe could be passed down in front of the meatus. Considerable redness of the labia existed, with tumefaction extending up to the *mons veneris*. The adhesion was taken up by inserting a probe at the upper aperture and forcing it downward. The probe was then introduced into the urethra without difficulty, but no flow of urine occurred on its withdrawal. On a second insertion, and leaving it in a while, the urine flowed freely when it was withdrawn. The history of the case showed that a day or two previously the child had suffered pain, and febrile symptoms had existed, for which it had been given calomel. He inferred that the retention was caused by the tumefaction in and around the urethra, and desired the opinion of members as to the nature and cause of the inflammation—whether erysipelatous or not.

Dr. J. G. Westmoreland desired to know if he had any means of knowing how long the adhesion had existed?

Dr. W. F. Westmoreland stated that the mother reported having discovered the condition a day or two previous. He supposed it was of short duration, as the adhesion was not firm.

Dr. Taliaferro supposed the case one of ordinary vulvitis, probably from want of cleanliness, allowing fecal accumulations, etc., within the vulva. He judged that the case was specific. Simple vulvitis often exists without any known cause, and runs on to serious eonsequences.

Dr. John M. Johnson thought that the case might have arisen from acid urine, as such cases, in his opinion, are often produced. Has a case under treatment of extensive ulceration of labia minora which he thinks originated from this cause. He also mentioned the fact that his experience taught him that ascarides, making their way from the rectum, produce excessive irritation in the vulva, and might lead to inflammatory excitement, such as the one reported by Dr. Westmoreland. He thinks the various cause of irritation by hand, ascarides and acid urine fruitful sources of the trouble.

Dr. W. F. Westmoreland stated that he thought the case different from ordinary vulvitis, the tumefaction was unusually great. Was in doubt whether the inflammation was erysipelatous in its character or not.

Dr. Taliaferro wished to know if there was any discharge of pus, and if there was appearance of abscess.

W. F. Westmoreland discovered only a slight mucous discharge—no abscess.

Dr. Taliaferro: Vulvitis in children, while not very common, is very apt to be grangrenous, the inflammation extending to the cellular tissue. No doubt the case was one of cellular inflammation—acute inflammation of vulva.

Academy adjourned.

